# Smart cities from low cost to expensive solutions under an optimal analysis

**DOI:** 10.1186/s40854-023-00448-8

**Published:** 2023-03-03

**Authors:** Romeo-Victor Ionescu, Monica Laura Zlati, Valentin-Marian Antohi

**Affiliations:** 1 Dunarea de Jos University, 111th, Domneasca Street, 800201 Galati, Romania; 2 Dunarea de Jos University, Nicolae Balcescu, Street, No. 59-61, 800001 Galati, Romania; 3 Department of Finance, Accounting and Economic Theory, Transylvania University, Brasov, Romania

**Keywords:** IoT, Smart city, Smart economy, Sustainability, Urban development

## Abstract

This scientific approach mainly aims to develop a smart city/smart community concept to objectively evaluate the progress of these organizational forms in relation to other classical/traditional forms of city organizations. The elaborated model allowed the construction of the dashboard of access actions in the smart city/smart community category on two levels of financial effort correlated with the effect on the sustainable development of smart cities. The validity of the proposed model and our approach was supported by the complex statistical analysis performed in this study. The research concluded that low-cost solutions are the most effective in supporting smart urban development. They should be followed by the other category of solutions, which implies more significant financial and managerial efforts as well as a higher rate of welfare growth for urban citizens. The main outcomes of this research include modelling solutions related to smart city development at a low-cost level and identifying the sensitivity elements that maximize the growth function. The implications of this research are to provide viable alternatives based on smart city development opportunities with medium and long-term effects on urban communities, economic sustainability, and translation into urban development rates. This study’s results are useful for all administrations ready for change that want the rapid implementation of the measures with beneficial effects on the community or which, through a strategic vision, aim to connect to the European objectives of sustainable growth and social welfare for citizens. Practically, this study is a tool for defining and implementing smart public policies at the urban level.

## Introduction

From a social point of view, the sustainable development of cities under the smart city—smart community concept is the most important objective, ensuring the social well-being of community members; this is an important point. This is why the research examines the best practices in sustainable development and smart city development. Simultaneously, the current scale of the pandemic is an additional argument for examining urban development efforts for smart city-smart community solutions.

The urban overpopulation and urban growth poles cause significant differences in social and economic development for these community members compared with other forms of population aggregation, and offer direct benefits to residents by sustainably increasing the quality of life and adaptability of the population to permanent societal challenges. In contrast, this urban agglomeration has amplified effects during the pandemic period through the accumulation of aggravating health and economic factors, but also during general periods through environmental pollution. In relation to the European strategic development objectives, the limitation in time of these effects is considered an aspect of sustainable development, the practices of limiting the adverse effects being supported by long-term financing by European and global organisms.

In this context, the concept of smart city has acquired a nuance and aroused the interest of researchers both theoretically and practically, currently existing top cities on all indicators that have approached the concepts of smart cities or smart communities to manage the negative aspects of urban agglomeration growth.

In this sense, a combination of scientific approaches shows that the implementation of smart management in communities can generate viable solutions to specific problems; however, there is need for an integrated approach to achieve the status of a smart city or community.

The literature contains various concerns related to smart city development, particularly regarding the effect of smart cities on quality of life, in conjunction with the identification of funding sources to support urban administrative activity in the direction of decreasing pollution, increasing the dynamism of sustainable development, and reducing environmental effects. Therefore, the literature stipulates several models based on the six pillars (smart city economy, smart people, smart mobility, smart environment, smart living, and smart governance), as well as the psychological perception of smart development. These aspects will be analyzed in the literature review section and motivate the following *research objectives*:

***O1:*** critical analysis starting from the current state of scientific knowledge in the field.

***O2:*** the development of the smart city/smart community concept to objectively evaluate the progress of these forms of organization in relation to other classical/traditional forms of town organization.

***O3:*** the transposition of this model by pivoting the hypotheses into a minimal and extended dashboard with pivoting links on each branch of the table facilitates the step-by-step implementation of this form of superior organization of urban populations.

***O4:*** the identification of simple solutions, implementable in the short term, up to extensive solutions with high efficiency and high value-added contribution to the life of the applicant city inhabitants.

The objective of this scientific approach is represented by a critical analysis starting from the current state of scientific knowledge in the field, the development of the smart city/smart community concept, to objectively evaluate the progress of these forms of organization in relation to other classical/traditional forms of town organization.

In accordance with the objectives discussed earlier in this study, the authors achieved statistical analysis of the socio-demographic and economic indicators reported through the Eurostat platform by the first 10 European smart cities. The city of Bucharest was added to this list as an anti-pole for smart city development.

The statistical data processed in dynamics allowed the consolidation of the smart indicators at the level of the selected sample (local Gross Domestic Product (GDP), Internet users, Research, Development and Innovation (RDI), Internet of Things (IoT), Internet banking, general motor rate, green vehicle motor rate, and polluting vehicle motor rate). These indicators were structured according to the average evolution of the selected sample during the period from 2009 through 2020. The main observations of the core indicators allowed the elaboration of a smart economic model, which analyzes the effect at the sample level of the adaptation of the smart city/smart community policies.

The elaborated model allowed the construction of the dashboard of access actions in the smart city/smart community category on two levels of financial effort correlated with the effect on the sustainable development of smart cities. The results of the study are useful to all administrations ready for change and that want the rapid implementation of the measures with beneficial effects on the community or which, through a strategic vision, aim to connect to the European objectives of sustainable growth and social welfare for citizens.

## Literature review

Scientific research shows great interest in smart urban development.

From the intersecting fields related to smart city points of view, issues related to the effect of smart city development and urbanization in relation to climate change, management, and urbanization are basic themes in scientific research.

**Fig. 1:**
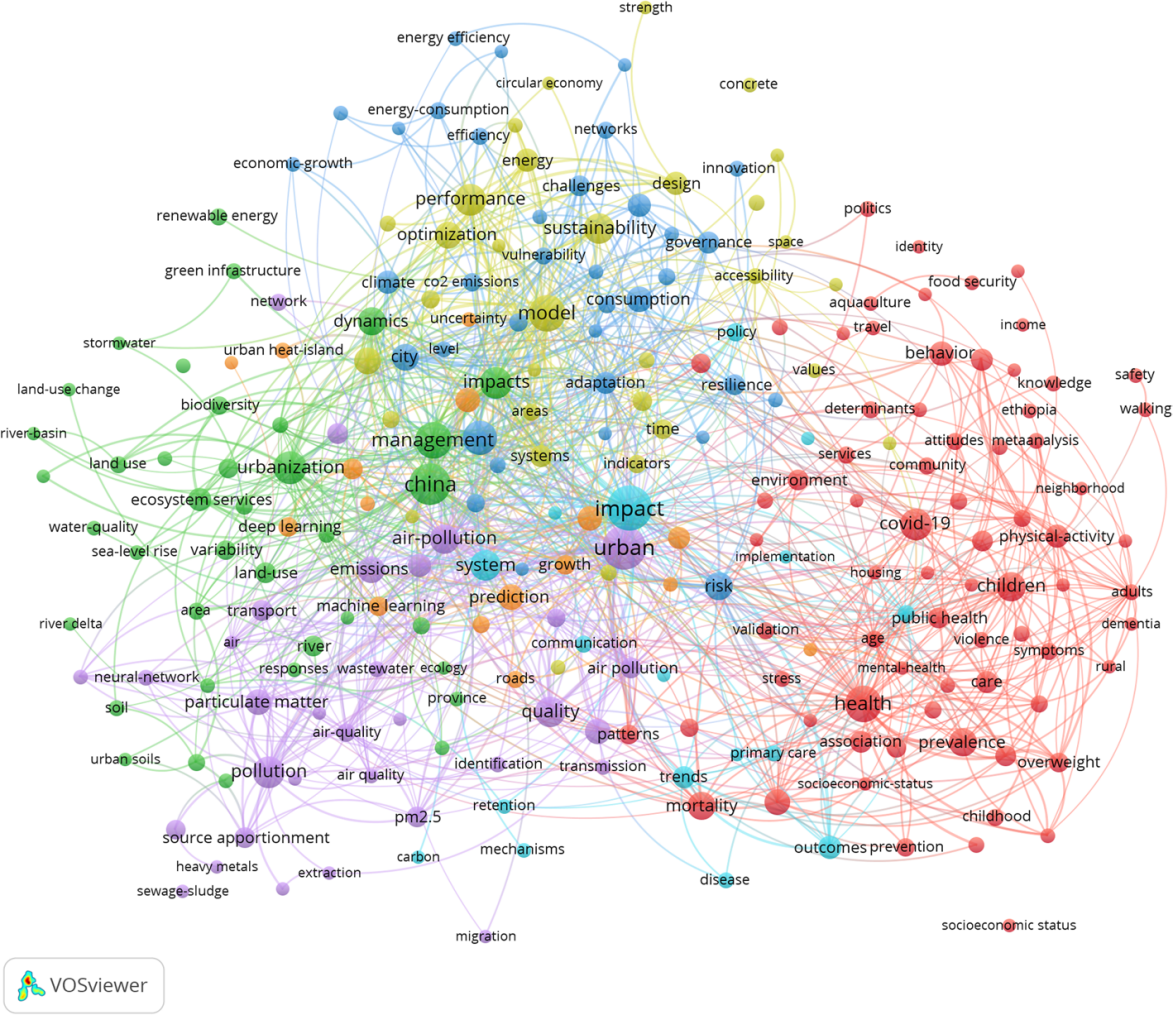
Map of smart city development research clusters. *Source*: VOSviewer

The diagram of connections in the research conducted using the VOSviewer software reflects the constitution of at least five significant clusters related to smart city development: health, environment, impact, urbanization, climate change, and pollution control clusters (see Fig. 1).

In support of this view, the authors Akande et al. (Akande et al. 2020) conduct a quantitative and qualitative bibliometric study based on 22 independent scientific studies, from which 249 dependency relationships on smart city development were extracted, relationships that aim to: behavioral control of the intention to promote social norms, attitude toward economic gain, risk perception, and other characteristics of specific smart city developments. According to this study, the most common observations are made in relation to subjective norms (trust and attitudinal factors), which together with risk perception and economic benefits, contribute to the intention to share. This model represents a closed smart development cluster that motivates the individuality and uniqueness of smart city development. From our point of view, the analysis should consider digitization issues, which were included in our research (Objective O1).

Different models have been developed to quantify smart-city capacity. One of them, a quadruple-helix model for smart city capacity measurement, directly connects the city and the innovation capacity of companies based on it. Consequently, the concept of a smart city is defined and analyzed using relevant phrases such as smart governance, smart economy, smart living, smart environment, smart mobility, and smart human capital. These concepts are derived from the smart use of strategy and planning, marketing, quality management, technological processes, logistics, and human resources Lombardi et al. (Lombardi et al. 2012). From our perspective, this approach has less of an effect on smart urban development. Consequently, a new and more efficient approach model was introduced (objective O3).

Smart urban development must be conducted under environmental protection and reduction in energy consumption. Urban demographic dynamics is another important element. In this sense, a European regulatory framework (directives and technical norms) already exists. This research comprehensively analyzes the concrete way of applying these regulations in the case of the large Mediterranean city in Italy (Palermo). The entire analysis is supported by the Urban and Environmental Building Code as Implementation Tool. This Code covered four areas and 20 articles. These areas include environmental sustainability and context appraisal, energy performance of the building envelope, energy performance of technical systems, and renewable energy systems. The result of this approach was a smart planning protocol that could create an instrument between cloud governance and smart tools for local urban administration (Riva Sanseverino et al. 2014). This approach, which presents the directions of smart development in a structured way, is consistent with our research objectives O1 and O3.

Smart city services are treated in an aggregate manner and cover initiatives for communities with special needs, such as jobless, elderly, female, and disabled; vocational training services; heritage provision and protection services and economic support (e.g., loan provision for settlement); several types of consulting services (e.g., training and new business registration); volunteer and cultural activities; and Metro-WiFi infrastructure and pet-related services as they are both considered related to local quality of life (L. G. Anthopoulos & Reddick 2016). From our perspective, smart urban development would be difficult without this integrated approach and without simultaneously pursuing the well-being of citizens and the interconnection of administrative and urban aspects. In developed cities of the world, with obvious smart urbanization, this approach is functional and enables urban progress (objective O2).

From the economic and the quality of urban life perspectives, another study examines the costs of implementing the smart city concept, on their efficiency from the perspective of energy savings, production value, ease of implementation, improving quality of life, and others. The authors of this study discuss complex concepts, such as improving urban economic development and social acceptance (L. Anthopoulos & Giannakidis 2017). We believe that cost issues are reflected most significantly in smart city disparities. Therefore, urban agglomerations with sufficient resources and where GDP/capita is higher can achieve social acceptance of change more easily, while public administration management does not face major decision problems that would slow down development to ensure the current functionality objectives (objective O2).

An interesting concept in smart development is the energy dimension of a smart city. This allows a new understanding of urban systems and progress toward a new style of urban management. New urban management is based on energy and technology (Lai et al. 2016). We agree with this suggestion. We also conduct such research when conceptualizing the proposed model (objectives O3 and O4).

The technological dimension of a smart city can move the urban system in its spatial and temporal development. It must be completed using energy because cities represent the greatest energy consumers. Consequently, a new approach to the planning of a smart city must be based on the energy dimension. This approach was implemented in Italy, where concerns about city energy were real. International researchers used three dimensions to analyze the smart city concept: ECOware: natural capital; water/energy; and SMART environment. The concept of ECOware: natural capital includes hand-made capital, economic and financial capital, institutional capital, human capital, and social capital. Water/energy is focused on transport, business, city services, citizens, and communication. Finally, the SMART environment addresses SMART mobility, SMART economy, SMART governance, smart people, and SMART living (Lai et al. 2016). Some of these concepts were addressed without sheep, framing ourselves in the current development trends governed by the energy, geopolitical, and health crises (objectives O3 and O4).

An interesting analysis is that which connects accessibility by collective transit and the availability of space for parking vehicles following a smart approach. This accessibility considers two elements: individual motorized and collective and non-motorized transport accessibility (Gerundo et al. 2016). In a congested city, the need for parking spaces and traffic calming is significant. Accordingly, the aggregate components of the smart methodology are sufficient (objective O2).

The concept of a smart city is defined in connection with urban innovation, e-government, and human welfare. This connection represents the basis for defining and implementing new planning and management principles and ideas for smart urban governance (Anthopoulos 2017b). We consider this approach timely, aimed at clarifying some effect aspects of smart urban development in connection with the quality of life (objective O2).

The theoretical approach of the smart city concept and its evolution is made in reference work that aims to find answers to questions related to the type of technologies used by the smart city, the types of services offered by the smart city approach, the solutions offered by the smart city approach, and the differences between theory and practice in the case of smart cities. Moreover, the study analyzes the processes that synthesize a smart city project, how to mobilize resources, and the life cycle of the smart project (Anthopoulos 2017a). We note the timeliness of the approach, which is consistent with the methodology used in conceptualizing the model (objective O3).

The contemporary economic approach cannot be implemented without internet platforms. If the research discusses Industry 4.0, it can also discuss the IoT, which is connected to digital productive capacities. The main global economic actors already have industrial platforms 4.0: Germany has the “Industry 4.0 Platform,” China the “Made in China 2025,” and the USA has the “Industrial Internet Consortium.” The Industry 4.0 platform is connected to industrial manufacturing and IoT applications for healthcare management, transportation, energy, and smart cities (Sendler 2017). This approach is now outdated as society moves to a new level: Industry 5.0.

An interesting component of a smart city is education, and smart learning is a key element for innovative and sustainable urban development. Education can also promote creativity and innovation. Moreover, new concepts such as smart technology, smart teaching, smart education, smart e-learning, smart classrooms, smart universities, and smart society have been developed based on education. In this approach, the smart learning covers both traditional and informal learning, as well (Liu et al. 2017). In our opinion, education is a basic pillar that generates smart development, including that at the urban level (objective O2).

The Netherlands is among the main promoters of smart approaches. Accordingly, the Netherlands has advantages over other states. At the urban level, there is good collaboration between the local administration, the private sector, and the academic environment. Additionally, smart solutions are already being implemented on large scale in society. This process is also supported by the presence of important multinational companies in the country. The Netherlands also has an extremely high-performance physical and digital infrastructure, functional test stands, and strong civil society (Bamwesigye & Hlavackova 2019). This study uses components from the above approach to define the objectives and the proposed analysis model (objectives O3 and O4).

In Romania, large companies such as ENEL have launched smart programs at the urban communities level. This company's smart approach targets multiple connections between underground infrastructure, energy and transportation infrastructure, urban energy flows, personal urban flows, buildings, and green and public areas. The company's smart solutions, included in the ENEL X project, allow urban administrations to use a single interface and process interconnected and integrated services (EnelX-SRL 2021). In the context of the current global energy crisis caused by the war in Ukraine, energy solutions have become vital for smart urban development and for improving the clarity of citizens’ lives. Alternative energy solutions are a potential source for smart city development (objectives O2 and O3).

An interesting approach is to begin with the effect of urban development on the environment. This approach supports the construction of the ISUT Model. The model defines a composite index that can quantify the sustainability of urban development using a vector cartography of urban land and building an alpha numerical database. This database prepares the model’s inputs regarding green spaces and built-up areas (Gerundo et al. 2016). This approach can be considered interesting and timely.

A previous study presents different understandings of the smart city concept (Sun et al. 2016). This research considers the three theoretical dimensions of the concept: human, technology, and organization, between which, as an economic-financial flow, sharing services operate, which is the enabling factor for urban smart development. The two pillars of blockchain (sharing services and computing) interact with individual human and technological components. This approach was also used in our present scientific approach (objective O3).

Another interesting approach defines a smart city as a building based on six concepts: the smart city economy, smart people, smart mobility, smart environment, smart living, and smart governance (Vinod Kumar & Dahiya 2017). The validity of this approach has been confirmed in other scientific studies in the field.

Smart city development must be realized under sustainability. This approach began from the idea that a smart city represents an application of the IoT. Moreover, city development must improve the environment, citizen lifestyles, and governance performance. The basic approach is to develop smart technologies with minimal human interactions (Silva et al. 2018). This indicator (IoT) was also used in our modelling (objective O3).

A more optimistic approach considers the smart city a solution for rapid urbanization. Classic urban development is limited and is facing new challenges. Consequently, smart city development can face all new challenges, including pandemics (Okai et al. 2018). We assert that the problems caused by the pandemic have shown that in urban agglomerations, measures to fight the disease have become a support for digitization and urban progress.

The real advantages of a smart city are related to citizens’ life standards, local economic development, transport and traffic management, environment, and better connection to public administration. Under these new challenges, a smart city must be analyzed in terms of specific aspects: smart mobility, smart living, smart environment, smart citizens, smart government, and smart architecture in their connections to technologies and concepts (Ismagilova et al. 2019). We agree with this suggestion.

Following an extensive meta-analysis process, a study (Camero & Alba 2019) has highlighted the positive dynamics of the Smart City concept. Although the research in the field is extensive, there is no unified approach to this concept. The analysis of the database related to these approaches shows that developed areas such as Europe, North Africa, America, Australia, and Asia (especially China) are more focused on the implementation of this concept, which became a pole of interest for researchers since 2010, following the development of Industry 4.0. Modelling is interesting but has shortcomings related to the level of approach and timeliness of the research.

A new approach is interesting in that it introduces the concept of a smart ecosystem as a complex connection between governance/administration, transportation, agriculture, logistics, maintenance, education, and healthcare. Such a smart city has to be connected to Information Communication Technology (ICT), which can even improve the traditional urban infrastructure (Ahad et al. 2020). From our perspective, the analysis has been oriented toward other indicators that are considered more representative. However, we cannot deny the importance of the research conducted by these authors.

The goals of urban sustainable development are analyzed to quantify the quality of life. To realize this, the authors combine public resources, human and social capital, and information and communication technologies. The analysis is based on a survey implemented in Brazil and uses the application of structural equation modelling (SEM). The authors indicate that they succeeded in improving the governance of smart cities to enhance the quality of urban life (De Guimarães et al. 2020). This approach is among the perspectives of smart development. However, it must be framed in the overall context of digitization.

The Chinese experience in developing smart cities is presented in a study that covers 35 smart cities using principal component analysis and back propagation neural network techniques. Such a neural network considers six dimensional factors and 22 operating indices. According to the analysis results, the main element in the development of smart cities is the development of a smart economy through sustainable productivity in innovative companies and high-tech industries (X. Li et al. 2019). Our research does not consider urban agglomerations in China viable smart models for the EU because the specificities of urban development are different in Europe.

A real link between the smart city components seems to be the sustainable transport. Nowadays, almost all urban transport systems are unsustainable. Consequently, sustainable transport, including cycling, and the greater implication of women in this industry are analyzed starting from good practices in the Netherlands and Germany. The same analysis was conducted in Kenya and Uganda (Bamwesigye & Hlavackova 2019). Our research also addresses the need for sustainable urban transport and introduces specific indicators (objectives O2 and O3).

A review of the literature on smart governance and its implications for supporting active cooperation between citizens and public administration in developing smart cities was conducted in 2019. The authors considered smart development to be more theoretical than practical. Consequently, they proposed a new way of cooperation and an agenda that could improve the active partnership between citizens and public administration for smart development (Tomor et al. 2019). We used this research method to define the objectives and needs of smart urban development modelling (objective O2).

Many local governments struggle to eliminate different administrative or cultural barriers in their areas, trying to develop Smart Cities not for themselves but for their citizens. To do this, one of the solutions is clearly the possibility of gathering relevant actors from the local level, such as education organizations, businesses, and public administration institutions, and supporting them to work on common objectives. It is crucial to identify the critical stakeholders that could have a powerful direct and indirect effect on the design, implementation, and functionality of smart city institutions and mechanisms. Functional congruency refers to the match between stakeholders’ expectations regarding the implementation of a social responsibility code and their perceptions of how an organization or system is assessed from a social perspective (Cristache et al. 2019). We agree with the authors' approach regarding the implementation of social responsibility.

Yan et al. (2020) proposed an evaluation system based on self-assessment in a critical approach conducted through a study on the Chinese smart transport system that includes qualitative and quantitative dimensions. This study is based on the unique cellular development of smart infrastructure, incorporating the Information and Communication Technology (ITC) section, development and support mechanisms, and regulatory mechanisms within the smart city. We agree with the authors' statement that a smart transportation system is not analogous to a smart city system. In arguing our opinion, Cirillo et al. (2020) show that IoT data managers partially contribute to the challenges of the transport system, of which the most accentuated are in the area of environment and health. Consequently, the transport system constitutes the infrastructure for the smart city, but is also an integrated service whose data sources are collected from at least 13 distinct urban areas (facilities, parking, environment, fleet, etc.). Our research also uses IoT and ITC indicators in the analysis.

A comparative analysis of smart city development models in Europe, USA, and Canada in reality with Latin American countries shows the effect of economic disparities as real barriers in replicating development models (Bruno and Fontana 2021; Charnock et al. 2021; Leon and Romanelli 2020; Marchetti et al. 2019). However, considering the main pillars of development, it is safe to conclude that there are four main directions (economic, political, environmental, and social) that influence the development of the cities. The smart city portfolio includes an integration of the sustainable use of natural resources (smart environment), transport infrastructure (smart mobility), living conditions (smart living), social equity (smart people), competitiveness (smart economy), and administrative autonomy (smart governance). We agree with this approach and consider it a starting point in the analysis of the development of a smart city. In defining the model proposed in this study, we introduce indicators of the smart economy, smart governance, and smart mobility (objective O2).

Through economic development, information technology, and artificial intelligence, it is believed that quality (sustainable) added value can be created to counteract the negative effects of economic crises, especially in conflict zones, to achieve long-term development of the smart economy (Jiang 2020). Considering that the economy is a significant component of a smart city, we consider the findings of this study to contribute to the comprehensive dimension of smart development systems.

Setijadi et al. (2019) developed a smart city assessment model for readiness from a social perspective. The authors corroborate the smart variables in a structured neural model that integrates the influence of social perception with the contribution of net benefit. The conundrum is that smart city readiness implies acceptance of the current use of new technologies so that, consistently and continuously, the master plan can be prepared and implemented. Regional influencing factors were identified and rigorously tested through 20 hypotheses, promoting the idea of a reconsideration of technological utility through the prism of social and governmental perception in complementarity with net benefit. This approach is interesting despite having been transposed to the social perception segment, because it covers the motivational area without which the smart city concept cannot work.

Consistent with Mohseni (2021), a study Lima (2020) demonstrates that the social dimension represented by communities with similar concerns in the use of digital technology, infrastructure, and common wealth can function as a smart city, creating opinion corridors for faster development of these categories. The basic principle of creating smart organizations is to develop a collaborative ecosystem based on sustainable local infrastructure in conditions of innovative development based on innovative solutions. We believe that creating examples of smart organizations in cities can encourage local communities to adopt local sustainable models of problem management that will drive smart city development over time.

Colding et al. (2018) proposed an analysis of the overuse of big data. The authors begin with the assumption that any biological system involves costs, costs that imply development, and development works according to the principles of thermodynamics. Consequently, the structure of the system becomes expansive, and there is an inflection point from which a shift is made from skill leadership to skill leadership (artificial intelligence). This example is of interest for determining the maximum threshold of the smart management solution, as this threshold triggers excessive costs that do not significantly improve the system.

A regional approach based on the Visegrad model is developed by Kézai et al. (2020) and comprises a transposition of clusters of a trilateral concept of smart economy based on two complementary pillars: smart environment and smart society. Based on the six pillars presented by Marchetti et al. (2019), the authors draw for the area under analysis the smart development diagram according to which the economic and social areas represent development poles, while the environment and mobility represent the most difficult aspects to homogenize to ensure the smart development of cities. These issues represent challenges for Eastern European countries in the coming period, as mobility remains high owing to low income/capita compared to the EU average, while environmental problems tend to worsen, as Eastern European countries are exploiting resources to the detriment of creating sustainable net value added.

In a structured focus area approach, the authors (Abu-Rayash & Dincer 2021; Fialová et al. 2021; Valencia-Arias et al. 2021) assessed the resilience of the smart city infrastructure in relation to new challenges of population growth and technological progress. The approach brings together eight broad areas (economy, society, environment, governance, infrastructure and transport, energy, and pandemic resilience) in an integrated model tested in 20 cities worldwide. According to this model, increasing smart energy (innovative energy systems for smart cities) will result in a proportional increase in GDP/capita at least doubling the initial investment in smart energy. The probability diagrams extrapolated to the year 2100 show three scenarios of evolution. However, we consider that some of the effects at this extended horizon can be accounted for as having other possible causes, which induces skepticism on the approach, which can be considered novel and of interest in our scientific approach.

A documented approach based on critical modelling assessment of industry 4.0 and circular economy-related inputs highlights that improving the digital connection between waste management, pollution control, circular economy, and population health can support smart city development in a dependency matrix based on the smart healthcare waste disposal system (Chauhan et al. 2021; Kayikci et al. 2021; Kristoffersen et al. 2020). Although topical, the diversity of variables imposes a limitation on planning effects that the authors assume in the chapter on study limitations and future research.

Models for assessing the social component of smart cities in Middle Eastern countries involve a more pronounced dynamic of social indicators that can enhance or limit the development of smart cities (Mohseni 2021). Therefore, social concerns and the promotion of group interest may prevail over economic interest or competitiveness, limiting smart city development, while public–private partnerships and access to technology can reinforce the development of smart cities.

Smart education as an experimental basis for community development can be approached in a solution-problem manner through econometric models that quantify the level of acceptance of education in different learning environments (school, academic, lecture halls, and institutionalized education) (Sułkowski et al. 2021). This baseline assessment can contribute to a zero point in time to build strategies for the development of smart people in relation to disruptive factors generated by interaction with private space, domestic activities, educational platforms, and others.

A smart city is a concept of urban development that integrates technologies and systems to efficiently and securely manage intangible resources to further improve the quality of life, developing the community and protecting the environment (Cosmulese et al. 2021).

Hassan et al. (2021) proposed an analysis of the effects of IoT on a smart city. They critically analyzed the literature and showed that some segments, such as waste management, monitoring systems, intelligent water supply, urban irrigation monitoring, intelligent parking, or lighting systems are services on which the literature has established viable models with significant results of implementation and integration in the concept of smart city. In our opinion, it is necessary to analyze these achievements in the context of the global pandemic crisis, which has affected the major smart cities of the world, contributing to functional disruptions of already implemented smart mechanisms, and the role of IoT remains significant in the pandemic equation.

The contribution of the IoT to an energy-efficient smart and intelligent street road lighting system was presented by Chen et al. (2022). The authors indicate the superiority of the modern street road lighting system based on sensors and LED lamps compared with the classical one. This approach supports significant energy savings and sustainable use of energy resources. The main effects of this approach include a decrease in the consumption of energy and carbon emissions.

An interesting analysis of IoT in the context of device insecurity and low security on the Internet was realized by Zhang et al. (2020). The authors connect the big data necessary to make smart decisions to smart urban planning and propose a new approach, SafeCity, which can support efficient smart decisions across smart cities’ ecosystems. The decision process involves three steps, starting with data securing. The second step involves the computation of secured data, whereas the last step focuses on extracting visions from data. This approach is also connected to the SafeCity approach.

The adoption of blockchain technology to build a new digital smart city ecosystem represents a process analyzed by Singh et al. (2020). The analysis covers risk management and financial services related to cryptocurrency, the IoT, and social services. The authors indicate that artificial intelligence and blockchain technology allow the building of sustainable smart city ecosystems. The analysis in this study was supported by a consistent literature review.

A new approach regarding IoT was realized by Syed et al. (2021), who concluded that the technologies must be connected to human intervention. The main conclusion of this approach is that the IoT can induce comfort and productivity for citizens. However, the same authors showed that the deployment of IoT systems for smart cities faces mitigation measures.

An impressive meta-analysis was conducted by Wu et al. (2021), which covered 965 research articles from representative journals indexed by Scopus. To do this, the authors used VOSviewer, CiteSpace, and Carrot2. The first conclusion of this analysis was that ICT, smart power grids, and smart growth represent the strongest citations. The analyzed publications can be grouped into three clusters: information technology, energy, and environment; urban.

transportation; and urban policy, planning, and development. The main trend resulting from the analysis is related to the improvement in urban big data quality. Although the authors recognized the limitations of their analysis, which was important for the research approach.

The evolution of ICT and scientific research regarding smart cities was presented by Colding et al. (2018), who also pointed out the increasing complexity of these cities. This complexity is followed by an increase in energy consumption, which can affect the urban progress. The analysis considers the contradiction between city development, increasing energy consumption, and city management in the context of present smart cities.

Smart urban development under the effect of innovation financing and collaboration systems is the subject of other research Blanck and Ribeiro (2021). They analyzed young companies in 66 cities from Europe from 2008 to 2014 and highlighted the role of private financial support for higher education entities in finding smart urban development solutions. Moreover, the authors consider that public financing does not significantly influence the evolution of urban development strategies.

The smart city concept is modelled as a complex adaptive system that belongs to Khan et al. (2021). The authors consider services, resources, and citizens and identify four characteristics of contemporary smart cities: sustainability, urbanization, quality of life, and smartness. The analysis covers various aspects of smart urban development that target micro (firms in industry, commerce, and urban population), mezzo (power companies and utility providers), and macro (government administration generating public policies and strategic public policy directions) levels. The analysis concludes that there are multilevel solutions for both mega and smart cities that are still under development. However, these solutions can improve even through partial implementation of the life of city citizens.

Rapid global population growth requires researchers to consider resilient structures capable of developing innovative energy systems in smart cities (Abu-Rayash and Dincer 2021). The analysis focuses on the differentiation of urban development levels. According to the smart city index, the top smart cities are Montreal, New York, Vancouver, Halifax, Osaka, and London. At the opposite pole are the cities of Abuja, Amman, Addis Ababa, and Tunis.

The realization of a sustainable urban transport network is supported by the deployment of electric vehicles. Authors such as Kou et al. (2022) promoted the idea of using solar-charged electric vehicles in the context of the massive promotion of solar energy projects based on the principles of group decision making (GDM) and spherical fuzzy numbers. The analysis provides solutions to the effectiveness of solar energy projects, design of solar panels with flexible structures, and so on.

Group decision-making (GDM) is approached from the perspective of the number of people involved and the heterogeneity of information by Li et al. (2022). To solve the challenges that arise in this decision-making process, the authors proposed a fuzzy cluster analysis and cluster approach. This approach aims to achieve an optimal consensus solution in small groups and then form a unified opinion.

The development of smart cities implies a decrease in costs, a high supply of high-quality services, and an increase in client satisfaction in achieving a financial system. Kou et al. (2021) analyzed the Fintech-based investments of European banking using interval type 2 (IT2) fuzzy decision-making, IT2 fuzzy testing and evaluation lab, and TOPSIS IT2 fuzzy models. The results of the analysis support the idea that European banks need to focus mainly on payment and money transfer alternatives to attract customers' attention and meet their expectations, while lowering costs.

According to the above literature review, it is clear that, in dynamics, the concept of smart city has gained consistency since the '00 s, when against the background of increasingly obvious climate change, complemented by changes in demographic trends (in the sense of their accentuation) and marked economic fluctuations deep crises, which destabilized the global economy) was the problem of organizing large cities in the sense of fruitful technological and digital development.

Therefore, the components of the main elements of local economic development were monitored in accordance with the increasingly stringent environmental and resource protection objectives. The core of monitoring smart city development is composed of capital, energy, and water resources, and an environment with applicative purposes at the level of local economic development on smart components. The synthesis of this desideratum is the increase in the urban population’s quality of life and normalization of the risk factors associated with demographic growth at the global, national, and local levels.

In this structure, the opportunity for the study proposed by the authors is demonstrated. It aims to create a tool for the evaluation and gradual implementation of the smart concept starting from socio-economic indicators related to the monitoring core and the top 10 cities in Europe that have made significant progress in the smart city area.

## Methodology

**Fig. 2:**
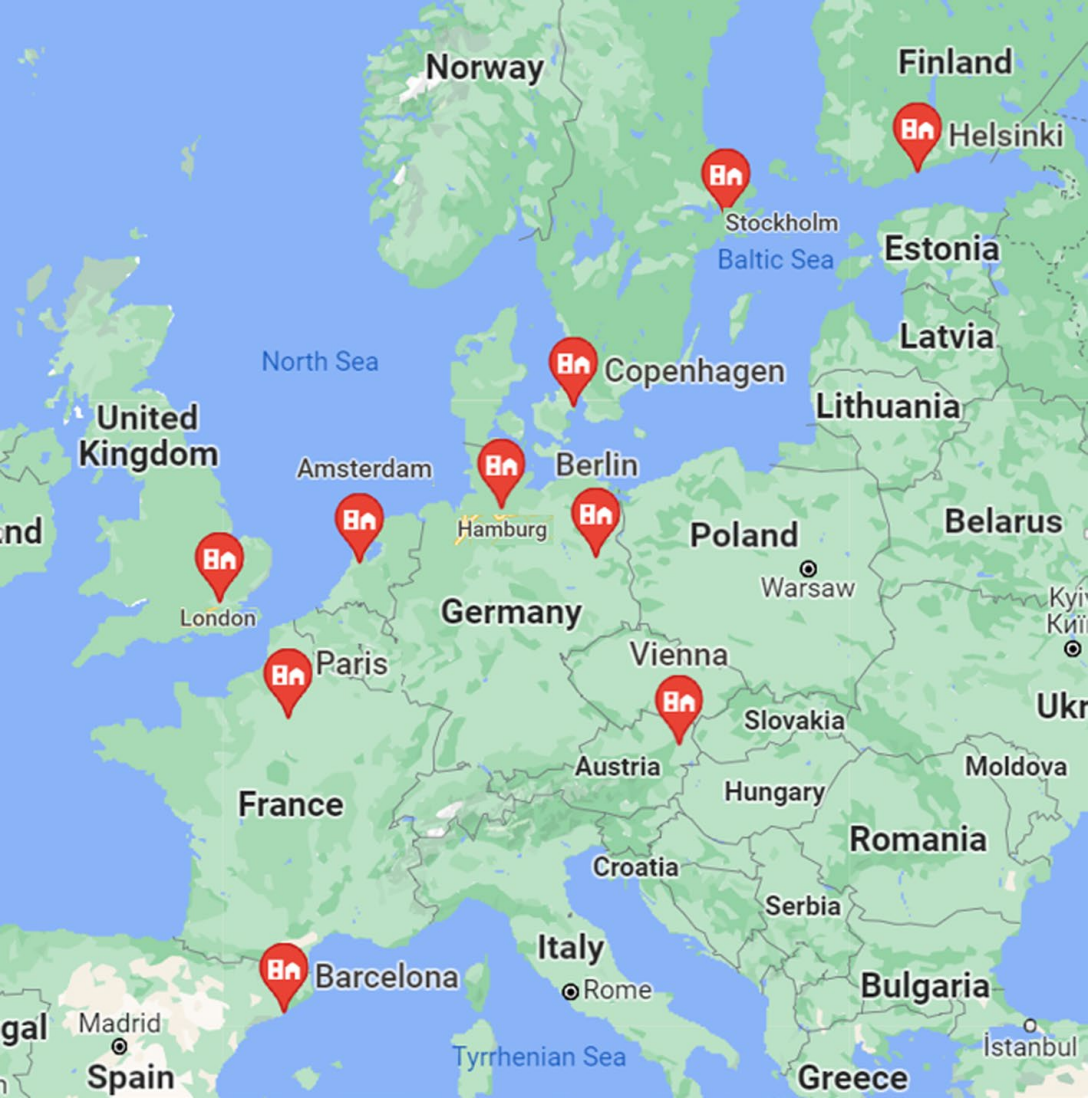
Top ten European smart cities in 2020. *Source*: Wilson (2020)

Several attempts have been made to classify smart cities in Europe. One of them (Wilson, 2020), realized by the Eden Strategy Institute and Ong & Ong, shows the following ranking: Copenhagen, Denmark; Amsterdam, Netherlands; Vienna, Austria; Barcelona, Province Barcelona, Spain; Paris, France; Stockholm, Sweden; London, United Kingdom; Hamburg, Berlin, Germany; Helsinki, Finland. Figure 2 presents a map of the first ten European smart cities in 2020.

These were incorporated as an anti-pole to the city of Bucharest, which stands out with similar demographic characteristics (2,583,460 inhabitants) in relation to the general average population of the 10 smart cities (2,265,876 inhabitants). Instead, the rest of the economic indicators were significantly lower than those of the evaluated smart cities in Bucharest. For example, GDP / capita is 0.02 million Euro / inhabitant in Bucharest compared with the average of smart cities of 0.04 million Euro / inhabitant.

In the analysis of this scientific approach, atypical "new" indicators compared with dedicated classical studies were used. However, with a significant effect on urban smart development. The indicators were as follows:

- *Local GDP* reflects the economic development of cities classified in terms of smart development and certified by the international top rankings made by the Eden Strategy Institute and Ong & Ong’s (OXD) research report.
- *Internet users* the urban consumption of Internet communications that attests to the possibilities of implementing intelligent communications among the population and its opening up to the assimilation of new technologies in the field.
- *RDI* the level of innovation reached or implemented at the local level as a factor of economic development associated with the acceptance of new technologies and the integration of these technologies in daily activities. This indicator is consistent with European objectives promoted through the competitiveness axis through technological R&D and innovation.
- *IoT* is a smart city-specific indicator that involves the use of the Internet to interconnect devices and services through network devices. In dynamics, the use of IoT started with the expansion of logistics chains to make them more efficient (2000s), reaching the interconnection of security networks and supervision of food transport and administrative services in 2010. Currently, it has reached the stage of monitoring and controlling remote objects through teleoperation and manipulation of services (tele school, tele work, tele health).
- *Internet banking* (banking) is an indicator of the financial capacity of the local market that evaluates the flexibility of the financial system used by the inhabitants and its capacity to connect to global financial flows in a safe and secure environment.
- *The general motorization rate* (CAR) is considered from the perspective of its contribution to local pollution. This indicator evaluates the urban transport capacity related to service sustainability. As the taxonomy of ecological transport services is recent, this general indicator can measure sustainable dynamics only in relation to the following two indicators:
- *Motor vehicle rate of green vehicles* (NEW_CAR_) calculated based on registrations of new ecological vehicles less than two years old;
- *Motorization rate of polluting vehicles* (OLD_CAR_), calculated based on registrations of polluting vehicles older than 10 years.

**Fig. 3:**
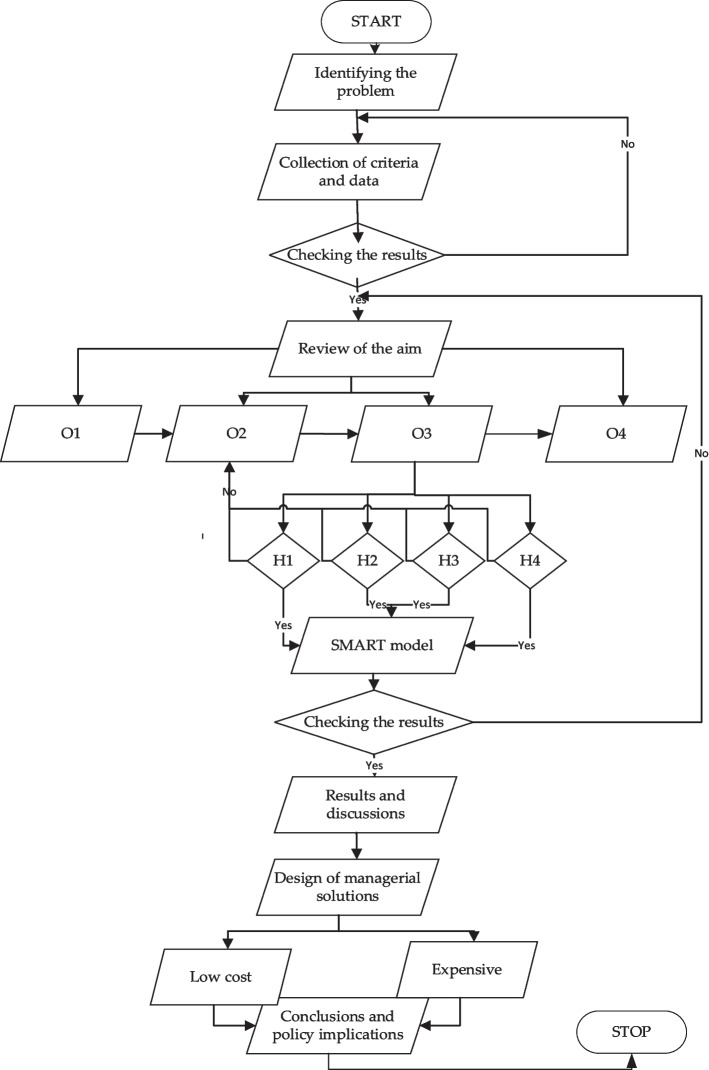
Study design

To achieve the research objectives, the following *study algorithm* was built to achieve the research objectives (Fig. 3).

To test the homogeneity of the data series collected from the Eurostat platform, the non-parametric Kolmogorov–Smirnov test was used, which demonstrated that by rejecting the null and the alternative hypotheses, the homogeneity of the sample is as follows in Table 1.

**Table 1:** Statistical analysis of the null hypothesis

Hypothesis test summary			
Null hypothesis	Test	Sig.	Decision
The distribution of Official population is normal with mean 2.294.747 and standard deviation 2,202,215.786	One-Sample Kolmogorov–Smirnov Test	0.029^a^	Reject the null hypothesis
The distribution of INTERNET-2016 is normal with mean 87 and standard deviation 7.148	One-Sample Kolmogorov–Smirnov Test	0.047^a^	Reject the null hypothesis
The distribution of INTERNET-2018 is normal with mean 89 and standard deviation 5.047	One-Sample Kolmogorov–Smirnov Test	0.041^a^	Reject the null hypothesis
The distribution of INTERNET-2019 is normal with mean 91 and standard deviation 6.235	One-Sample Kolmogorov–Smirnov Test	0.011^a^	Reject the null hypothesis
The distribution of IOT-2013 is normal with mean 73 and standard deviation 15.312	One-Sample Kolmogorov–Smirnov Test	0.047^a^	Reject the null hypothesis
The distribution of IOT-2014 is normal with mean 74 and standard deviation 14.588	One-Sample Kolmogorov–Smirnov Test	0.036^a^	Reject the null hypothesis
The distribution of Banking-2020 is normal with mean 74 and standard deviation 20.323	One-Sample Kolmogorov–Smirnov Test	0.035^a^	Reject the null hypothesis
The distribution of CAR-2009 is normal with mean 17.334.825 and standard deviation 16,037,305.994	One-Sample Kolmogorov–Smirnov Test	0.021^a^	Reject the null hypothesis
The distribution of CAR-2010 is normal with mean 17.528.774 and standard deviation 16,217,332.357	One-Sample Kolmogorov–Smirnov Test	0.022^a^	Reject the null hypothesis
The distribution of CAR-2011 is normal with mean 17.701.606 and standard deviation 16,395,452.280	One-Sample Kolmogorov–Smirnov Test	0.023^a^	Reject the null hypothesis
The distribution of CAR-2012 is normal with mean 17.882.952 and standard deviation 16,568,308.953	One-Sample Kolmogorov–Smirnov Test	0.023^a^	Reject the null hypothesis
The distribution of CAR-2013 is normal with mean 18.180.288 and standard deviation 16,826,907.303	One-Sample Kolmogorov–Smirnov Test	0.020^a^	Reject the null hypothesis
The distribution of CAR-2014 is normal with mean 18.298.673 and standard deviation 16.926156.882	One-Sample Kolmogorov–Smirnov Test	0.020^a^	Reject the null hypothesis
The distribution of CAR-2015 is normal with mean 18.488.342 and standard deviation 17,074,308.925	One-Sample Kolmogorov–Smirnov Test	0.021^a^	Reject the null hypothesis
The distribution of CAR-2016 is normal with mean 18.771.165 and standard deviation 17,276,098.823	One-Sample Kolmogorov–Smirnov Test	0.020^a^	Reject the null hypothesis
The distribution of CAR-2017 is normal with mean 19.063.598 and standard deviation 17,446,381.917	One-Sample Kolmogorov–Smirnov Test	0.019^a^	Reject the null hypothesis
The distribution of CAR-2018 is normal with mean 19.337.658 and standard deviation 17,616,522.414	One-Sample Kolmogorov–Smirnov Test	0.019^a^	Reject the null hypothesis
The distribution of CAR-2019 is normal with mean 19.588.177 and standard deviation 17,792,833.690	One-Sample Kolmogorov–Smirnov Test	0.019^a^	Reject the null hypothesis
The distribution of CAR-2020 is normal with mean 19.866.565 and standard deviation 17,981,294.029	One-Sample Kolmogorov–Smirnov Test	0.019^a^	Reject the null hypothesis
The distribution of NEW_CAR_-2009 is normal with mean 2.647.154 and standard deviation 2,444,367.156	One-Sample Kolmogorov–Smirnov Test	0.004^a^	Reject the null hypothesis
The distribution of NEW_CAR_-2010 is normal with mean 2.519.893 and standard deviation 2,341,374.842	One-Sample Kolmogorov–Smirnov Test	0.005^a^	Reject the null hypothesis
The distribution of NEW_CAR_-2011 is normal with mean 2.320.708 and standard deviation 2,154,252.829	One-Sample Kolmogorov–Smirnov Test	0.024^a^	Reject the null hypothesis
The distribution of NEW_CAR_-2012 is normal with mean 2.303.830 and standard deviation 2,173,046.909	One-Sample Kolmogorov–Smirnov Test	0.031^a^	Reject the null hypothesis
The distribution of NEW_CAR_-2014 is normal with mean 2.251.151 and standard deviation 2,172,256.564	One-Sample Kolmogorov–Smirnov Test	0.018^a^	Reject the null hypothesis
The distribution of NEW_CAR_-2019 is normal with mean 19.600.384 and standard deviation 17,802,107.469	One-Sample Kolmogorov–Smirnov Test	0.019^a^	Reject the null hypothesis
The distribution of NEW_CAR_-2020 is normal with mean 19.875.697 and standard deviation 17,988,234.697	One-Sample Kolmogorov–Smirnov Test	0.019^a^	Reject the null hypothesis
The distribution of OLD_CAR_-2009 is normal with mean 5,891,278.808 and standard deviation 5,406,678.465	One-Sample Kolmogorov–Smirnov Test	0.011^a^	Reject the null hypothesis
The distribution of OLD_CAR_-2010 is normal with mean 6,022,711.647 and standard deviation 5,452,717.427	One-Sample Kolmogorov–Smirnov Test	0.012^a^	Reject the null hypothesis
The distribution of OLD_CAR_–2011 is normal with mean 6,072,948.025 and standard deviation 5,471,075.665	One-Sample Kolmogorov–Smirnov Test	0.013^a^	Reject the null hypothesis
The distribution of OLD_CAR_-2012 is normal with mean 6,240,598.701 and standard deviation 5,555,250.104	One-Sample Kolmogorov–Smirnov Test	0.030^a^	Reject the null hypothesis
The distribution of OLD_CAR_-2013 is normal with mean 6.191.282 and standard deviation 5,512,597.718	One-Sample Kolmogorov–Smirnov Test	0.014^a^	Reject the nll hypothesis
The distribution of OLD_CAR_-2014 is normal with mean 6.303.108 and standard deviation 5,571,509.819	One-Sample Kolmogorov–Smirnov Test	0.026^a^	Reject the null hypothesis

Asymptotic significances are displayed. The significance level is .05

^a^Lilliefors corrected

The above homogeneity test proves that the database used is valid, allowing econometric modelling and validation of the working hypotheses.

To elaborate on the proposed model, the following *working hypotheses* are used:

H1

The rate of urban economic development is significantly and directly proportional to the efforts to implement the RDI results and the openness of the inhabitants in the use of the IT infrastructure for the management of financial flows.

H2

Urban economic development insignificantly influences the pro-ecological attitudes of the inhabitants. To activate this, administrative steps are required in terms of infrastructure and service networks.

H3

The development of IoT is directly related to the openness of inhabitants in the use of IT infrastructure for the management of financial flows.

H4

The smart maximization function is valid and adequate if and only if there is a minimum level α_i_ of each of the above indicators for an urban population correctly dimensioned according to the urban endowments and minimum level of economic capacity measured based on the rate of urban economic development.

Based on the above hypotheses, a ***smart economic model*** was developed to analyze the effect of adapting smart city/smart community policies at the sample level. The model was developed using the least squares method (OLS) with simple linear regression in the following form:

1 $$\left\{ \begin{gathered} SMART_{GDP}^{ \wedge } = \alpha_{1} * NEW_{CAR} + \alpha_{2 * IoT} + \alpha_{3} * RDI + \alpha_{3} * Banking \hfill \\ \quad \forall i \in N,\;i \ne 0,\;\exists i,\;so\;that\;\mathop {\max }\limits_{i \to 1} \left( {SMART_{{GDP_{i} }} } \right) \gg GDP_{i} \cup \alpha_{i} > 0 \hfill \\ \end{gathered} \right.$$

where SMART_GDP_ is the smart function of the cities’ economic development based on the transformation of administrative flows into smart flows using IoT; NEW_CAR_, RDI, Banking.

## Results and discussions

The model ensures the evaluation of the smart policies’ applicability/efficiency at the smart city/smart community level, starting from the development core of the first 10 European smart cities published in specialized tops, in contrast to smart development antipoles. Following the modelling, the model equation becomes

2 $$\left\{ \begin{gathered} SMART_{GDP} = - 6856E^{ - 6} * NEW_{CAR} - 127.897 * IoT + \hfill \\ \quad + 53.74 * RDI - 75.988 * Banking \hfill \\ \quad \mathop {\max }\limits_{i \to 1} \left( {SMART_{{GDP_{i} }} } \right) \gg GDP_{i} \cup \alpha_{i} > 0 \hfill \\ \end{gathered} \right.$$

The connection of model Eq. (2) with the model assumptions is given by system (3):

3 $$\left\{ \begin{gathered} H1:\bigcap\nolimits_{i = 1}^{n} {\left( {\begin{array}{*{20}c} {SMART_{GDP}^{ \wedge } } \\ {RDI} \\ \end{array} } \right) \ne \emptyset } ,\quad \alpha_{3} > 0 \hfill \\ H2:\bigcap\nolimits_{i = 1}^{n} {\left( {\begin{array}{*{20}c} {SMART_{GDP}^{ \wedge } } \\ {NEW_{CAR} } \\ \end{array} } \right) \ne \emptyset } , \quad \alpha_{1} \ne 0 \hfill \\ H3:\bigcap\nolimits_{i = 1}^{n} {\left( {\begin{array}{*{20}l} {SMART_{GDP}^{ \wedge } } \hfill \\ {IoT} \hfill \\ {Banking} \hfill \\ \end{array} } \right) \ne \emptyset } ,\quad \left( {\begin{array}{*{20}c} {\alpha_{2} } \\ {\alpha_{4} } \\ \end{array} } \right) \ne 0 \hfill \\ H4: \mathop {\max }\limits_{ i \to 1} \left( {SMART_{{GDP_{i} }} } \right) \gg GDP_{i} \bigcup {\alpha_{i} > 0} \hfill \\ \end{gathered} \right.$$

The sample for which the model was applied concerned information related to the following smart cities: Copenhagen, Amsterdam, Vienna, Barcelona, Paris, Stockholm, London, Hamburg, Berlin, and Helsinki. The antipole of smart development, Bucharest, was used for comparison.

**Fig. 4:**
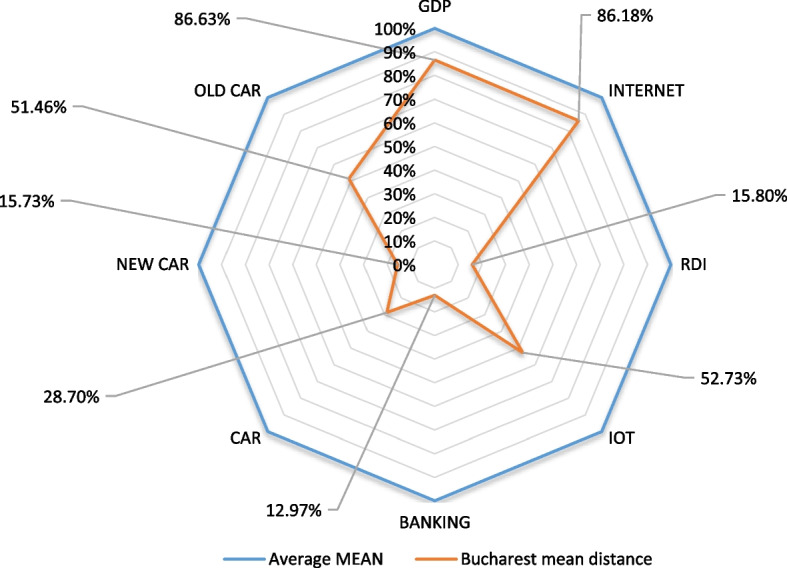
Presentation of the smart development at the evaluation date for the anti-pole of the model: the city of Bucharest

From the distribution average point of view, after applying the evaluation procedure on the frequency series pivoted after each evaluated smart city, we found that the antipole (Bucharest) is from the smart development point of view at a distance of 43% from the average evaluated sample, with differences from the sample (see Fig. 4).

Figure 4 clearly shows that, regarding the main components of smart development, the city of Bucharest is deficient in most key development points: RDI, IoT, banking, and new cars, but well represented by the use of the Internet and the engine rate of polluting vehicles. This aspect demonstrates the model’s usefulness and opportunity, which in the first step allows the evaluation of the development to identify the vulnerable points that need improvement strategies to maximize the smart function of the city.

The Pearson correlation table shows that, at the level of the analyzed sample regarding the concept of smart city integrated on modelled components (GDP, Internet, RDI, IoT, Internet banking, and motorization rates), the weakest correlation is found at the level of motorization of green vehicles, which is an active component of pollution reduction with a lower resilience to implementation, while Internet banking and the RDI component generate the most significant correlations on the Pearson correlation coefficient with sigma value tending to 0 (see Table 2).

**Table 2:** Pearson correlation

$\mathrm{\alpha }$	$\mathrm{\alpha }$	$\mathrm{\alpha }$	$\mathrm{\alpha }$	$\mathrm{\alpha }$	$\mathrm{\alpha }$	
$\mathrm{\alpha }$	$\mathrm{\alpha }$	$\mathrm{\alpha }$	$\mathrm{\alpha }$	$\mathrm{\alpha }$	$\mathrm{\alpha }$	0.652
	$\mathrm{\alpha }$	$\mathrm{\alpha }$	$\mathrm{\alpha }$	$\mathrm{\alpha }$	$\mathrm{\alpha }$	0.656
	$\mathrm{\alpha }$	$\mathrm{\alpha }$	$\mathrm{\alpha }$	$\mathrm{\alpha }$	$\mathrm{\alpha }$	0.584
	$\mathrm{\alpha }$	$\mathrm{\alpha }$	$\mathrm{\alpha }$	$\mathrm{\alpha }$	$\mathrm{\alpha }$	0.663
	$\mathrm{\alpha }$	$\mathrm{\alpha }$	$\mathrm{\alpha }$	$\mathrm{\alpha }$	$\mathrm{\alpha }$	1.000
$\mathrm{\alpha }$	$\mathrm{\alpha }$	$\mathrm{\alpha }$	$\mathrm{\alpha }$	$\mathrm{\alpha }$	$\mathrm{\alpha }$	0.011
	$\mathrm{\alpha }$	$\mathrm{\alpha }$	$\mathrm{\alpha }$	$\mathrm{\alpha }$	$\mathrm{\alpha }$	0.010
	$\mathrm{\alpha }$	$\mathrm{\alpha }$	$\mathrm{\alpha }$	$\mathrm{\alpha }$	$\mathrm{\alpha }$	0.023
	$\mathrm{\alpha }$	$\mathrm{\alpha }$	$\mathrm{\alpha }$	$\mathrm{\alpha }$	$\mathrm{\alpha }$	0.009
	$\mathrm{\alpha }$	$\mathrm{\alpha }$	$\mathrm{\alpha }$	$\mathrm{\alpha }$	$\mathrm{\alpha }$	

Using the entered method in the linear regression model (using the least squares method), a model in which the regression variable is local/regional GDP, as an implementation of the smart city concept’s favoring factor, all regression variables were included (NEW_CAR_, IOT, RDI, Banking). Consequently, no variables were excluded from the model.

The proposed model had a coefficient of determination of over 98% and a standard estimator error of 384.96%. The model is valid, with the sigma value of the change factor tending to zero, according to the summary of the model presented in Table 3.

**Table 3:** Model summary

Model^b^	R	R Square	Adjusted R Square	Std. Error of the Estimate	Change Statistics	
					R Square Change	F Change
1	0.995^a^	0.989	0.983	384.96550	0.989	159.245
Model	Change Statistics					
	df1	df2	Sig. F Change	Durbin- Watson		
1	4	7	0.000	1.665		

^b^Dependent variable: GDP mill. Euros

**Fig. 5:**
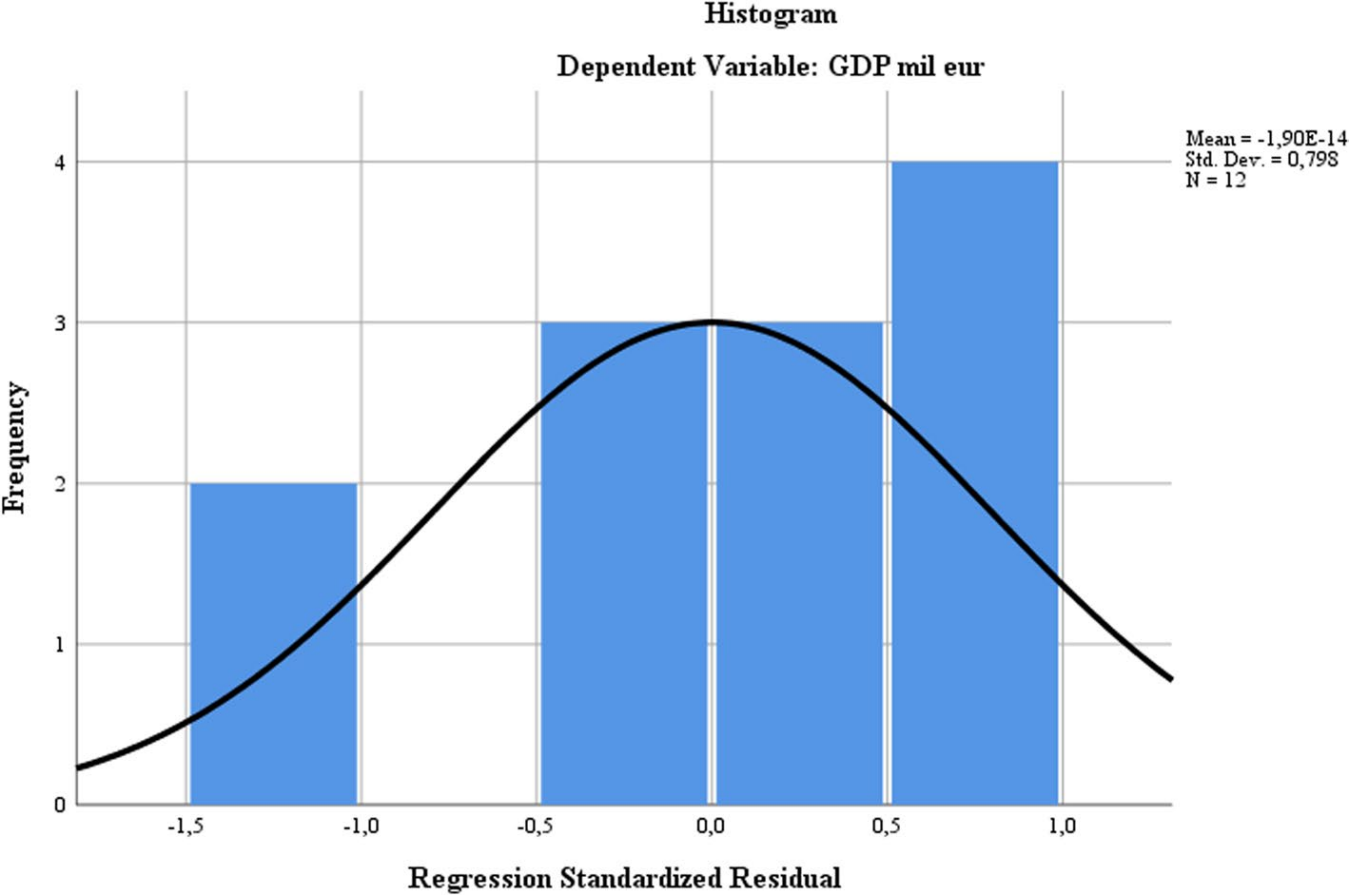
Normal distribution of the dependent variable. *Source*: Author`s contributions

From the normal distribution of the dependent variable, the histogram diagram reflects a homogeneous distribution with accumulation on the ascending slope (afferent to the anti-pole in the model) and on the descending slope. This demonstrates that there are significant differences between the distribution of the dependent variable for the analyzed smart city core in relation to the Bucharest antipole, which demonstrates that the evaluation is relevant and captures the differentiated smart development dynamics of the antipole from the rest of the smart city core in the sample (see Fig. 5).

Following the ANOVA test, it was found that the dependent variable in relation to the regressor variables was mainly characterized (98.91%) by the regression function, with the influence of the residual factor accounting for only 1.09%. The number of degrees of freedom in the regression function is four, and that in the residual function is seven. The value of the F test was 159.24 with sigma tending to 0 (see Table 4).

**Table 4:** ANOVA method

Model^a^		Sum of squares	df	Mean square	F	Sig
1	Regression	94,399,485.842	4	23,599,871.460	159.245	0.000^b^
	Residual	1,037,389.064	7	148,198.438		
	Total	95,436,874.906	11			

^a^Dependent variable: GDP mill. Euros

^b^Predictors: (constant), NEW_CAR_, IOT, RDI, Banking

**Fig. 6:**
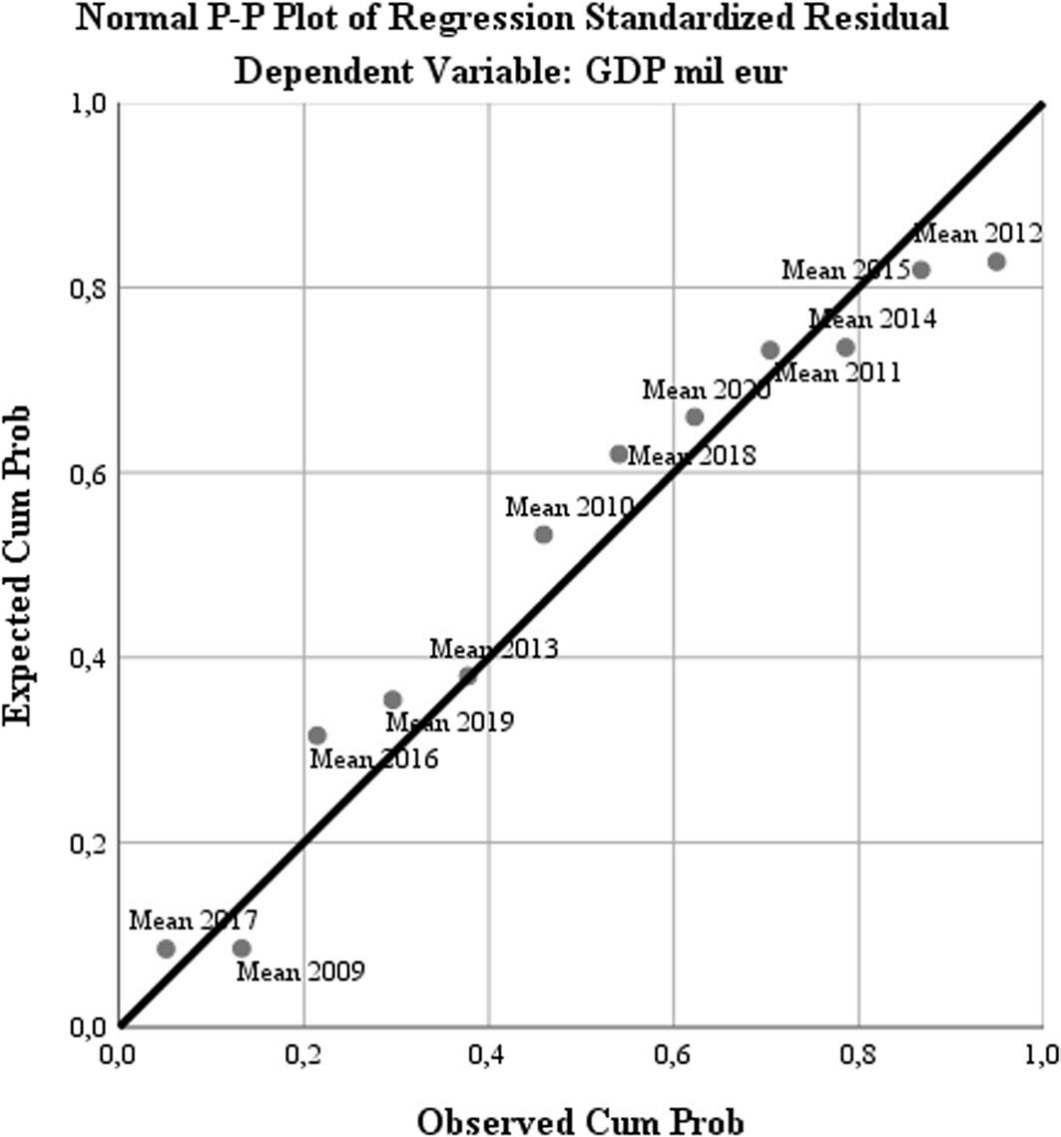
Normal P-P Plot of Regression Standardized Residual for Dependent Variable (GDP)

The P–P plot diagram for the presentation of the standardized residual regression for the dependent variable GDP reflects the fact that the years 2013, 2015, and 2011 are best represented for smart development according to the model, while the distance from the regression line that measures the average deviation of the observed values and the probabilistic evolution is maximized for the years 2010, 2016, and 2012. The years 2019 and 2020 show an average deviation from the forecasted evolution, indicating that the gradual development (in dynamics) of the cities and their transformation into cities with smart management is variable, with a sinusoidal development function and significant external influences induced by the macroeconomic context and the strategic development directions adopted by the EU associated with their financing (see Fig. 6).

Clearly, in relation to the model variable, the R&D function plays a significant role and is directly proportional with the dependent variable of the model, which demonstrates H1 of the model.

From the variable rate of motorization rate of green vehicles, there is an inversely proportional evolution of the variable in relation to the dependent variable at the level of representation of non-standard beta coefficients (-6,858 × 10^–6^) and at the level of correlation coefficients on the covariance matrix, proving H2 of the model.

The use of IoT has an inversely proportional correlation with the dependent variable, indicating that it is a managerial component with significant resource consumption, which has long-term performance in smart functions, and is dependent on openness to the use of non-polluting means and with the concept of R&D. According to the correlation of the coefficients, the IoT concept varies inversely with the Internet banking flow, which is a decelerator of IoT development. This is demonstrated by hypothesis H3.

The proposed model is homogeneous, well represented and significant for evaluating the smart development of cities in relation to a core of development poles in Europe and demonstrates H4 by comparing it with the Bucharest anti-pole.

Table 5 presents the correlation coefficients used in the argumentation of the hypotheses and their validation.

**Table 5:** Coefficient correlations

Model		NEW_CAR_	IOT	RDI	Banking
Correlations	NEW_CAR_	1.000	0.371	0.117	− 0.262
	IOT	0.371	1.000	0.021	− 0.303
	RDI	0.117	0.021	1.000	− 0.956
	Banking	− 0.262	− 0.303	− 0.956	1.000
Covariances	NEW_CAR_	6.219 × 10^–10^	0.001	7.558 × 10^–5^	− 0.002
	IOT	0.001	17,293.651	71.962	− 10,640.291
	RDI	7.558 × 10^–5^	71.962	669.097	− 6604.536
	Banking	− 0.002	− 10,640.291	− 6604.536	71,294.709

**Fig. 7:**
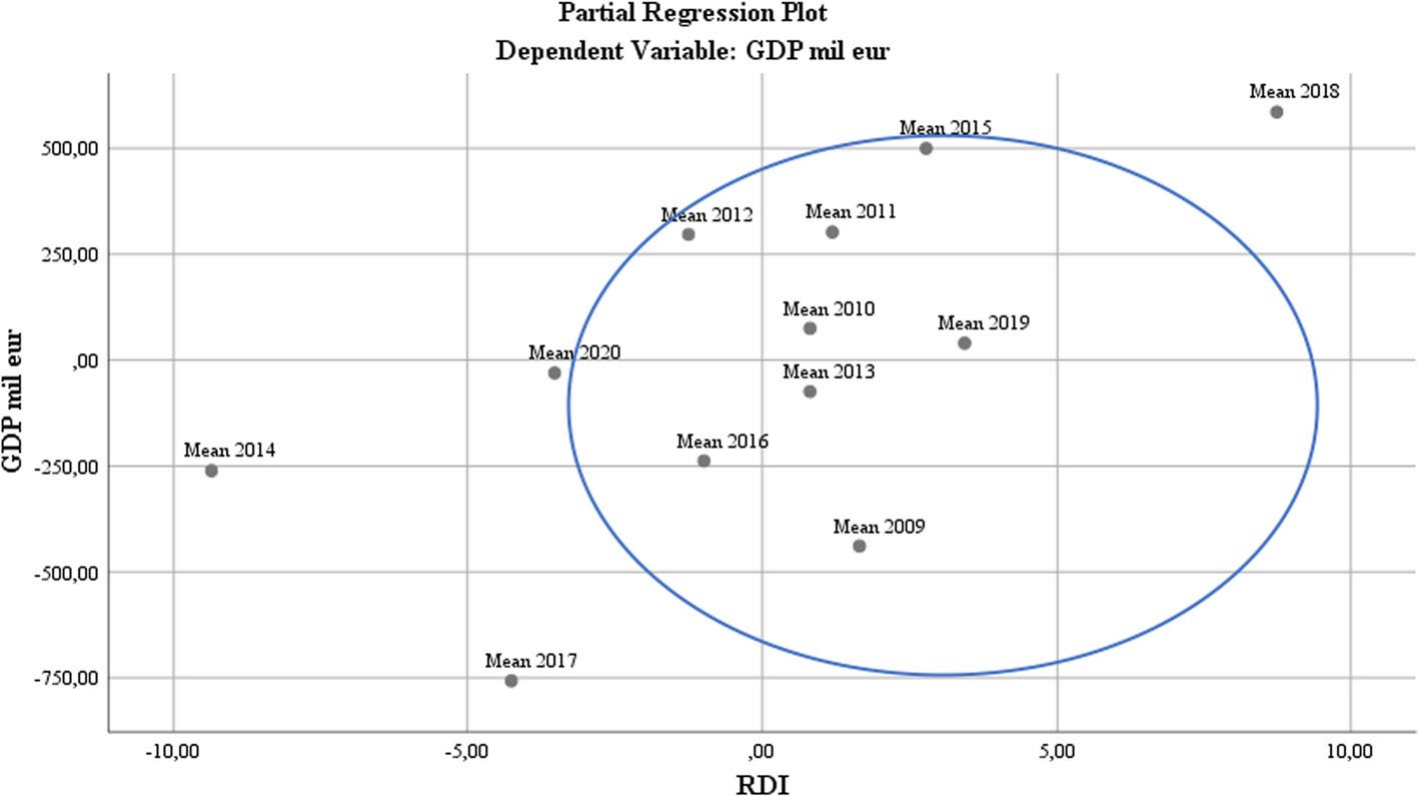
RDI regression analysis

At the level of partial regression on the regression variables of the model, the aim was to evaluate the development of these components as part of smart policies in dynamics (by years) at the level of the study sample. Regarding RDI, it was found that there is a unified practice regarding the coherent application of policies to approximate RDI policies as a smart part, except for 2014, 2017, and 2018. This evolution represents a logical consequence of the economic recovery processes at the EU level after global economic contractions owing to the crisis. Additionally, for 2017–2018, the initiation of Brexit was a difficult test for the European economy, a fact reflected in the table above by the presence of London (see Fig. 7).

**Fig. 8:**
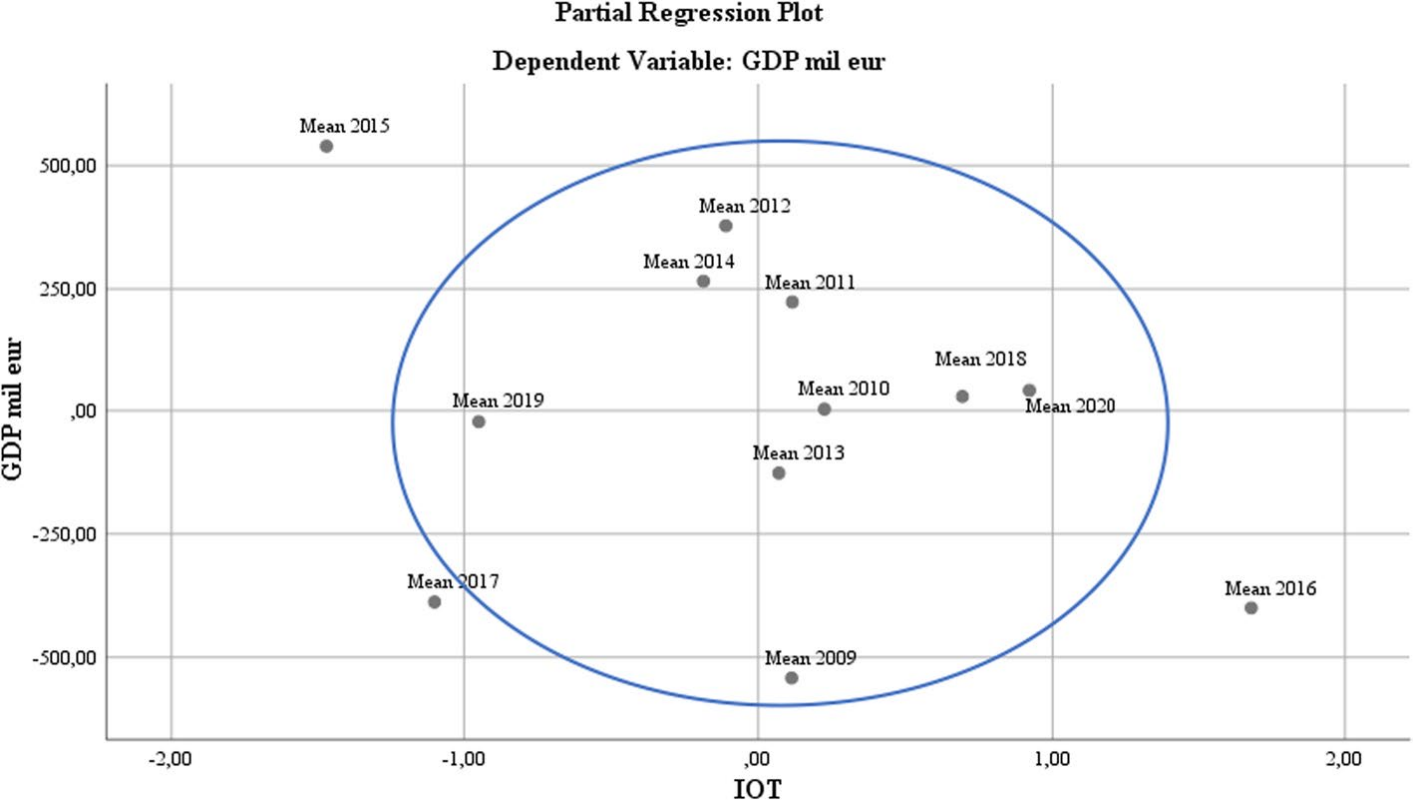
IoT regression analysis

At the IoT development level, there is a core focus on the entire period, excepting 2015 to 2017, where the incidents related to the Mirai malware (Munro 2021) https://www.pentestpartners.com/security-blog/what-is-mirai-the-malware-explained/ caused infections of digital video recorder devices, materialized in distributed denial-of-service attacks (in 2016, the KrebsOnSecurity block (Goodin 2016) was attacked with a 600 Gb stream, and in 2017, the Hajime worm infected many devices, blocking access to their ports), (see Fig. 8).

As the financial crisis evolved into the Eurozone debt crisis, more comprehensive integration of the banking system became clear. Initially, mechanisms were introduced: a single supervisory mechanism (SSM) (European Commission 2021b) and a single resolution mechanism (SRM) (European Commission, 2021a), which directly influenced the development of Internet banking. In October 2017, these two mechanisms became fully operational, resulting in a significant increase in unionist banking architecture in the EU, including the explosion of banking operations (see Fig. 8).

**Fig. 9:**
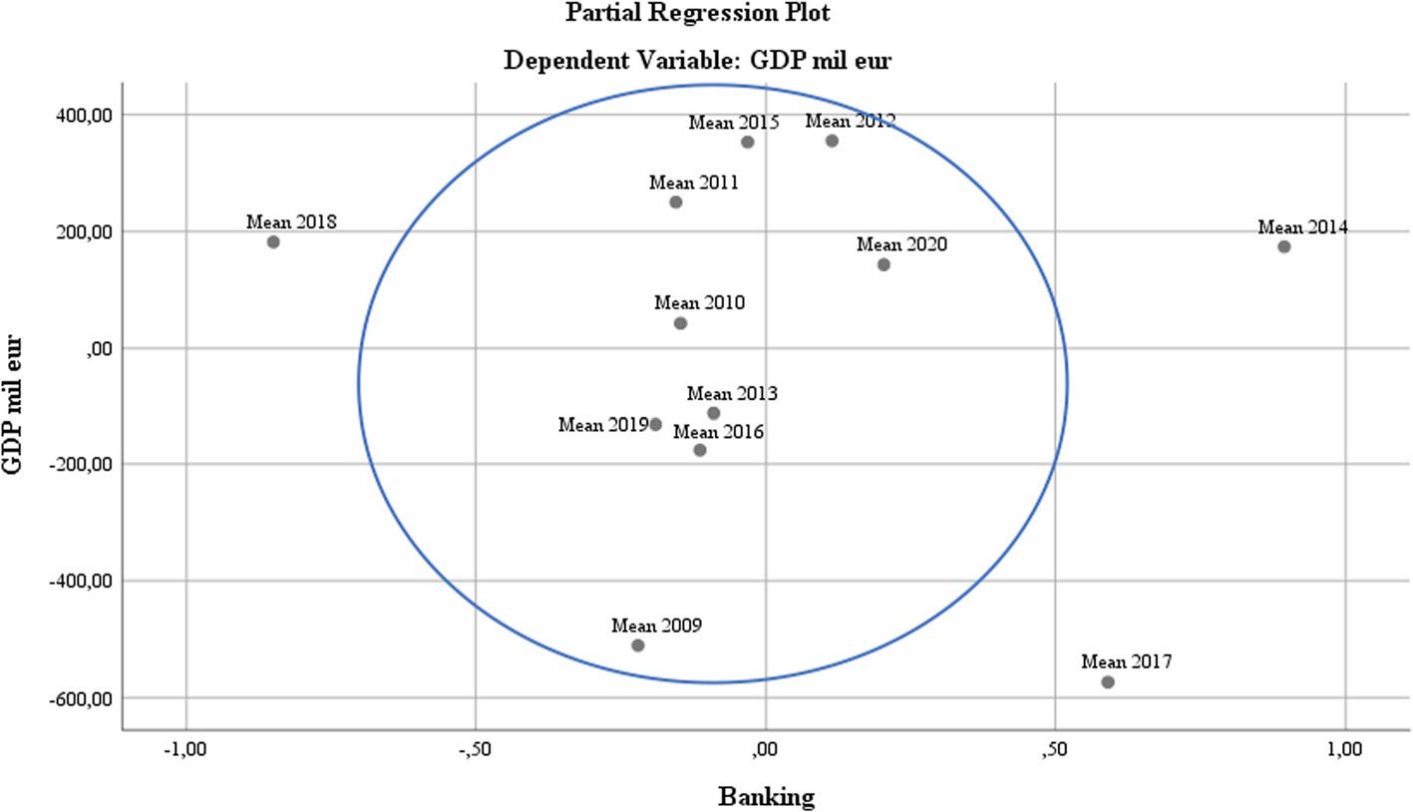
Banking regression analysis

**Fig. 10:**
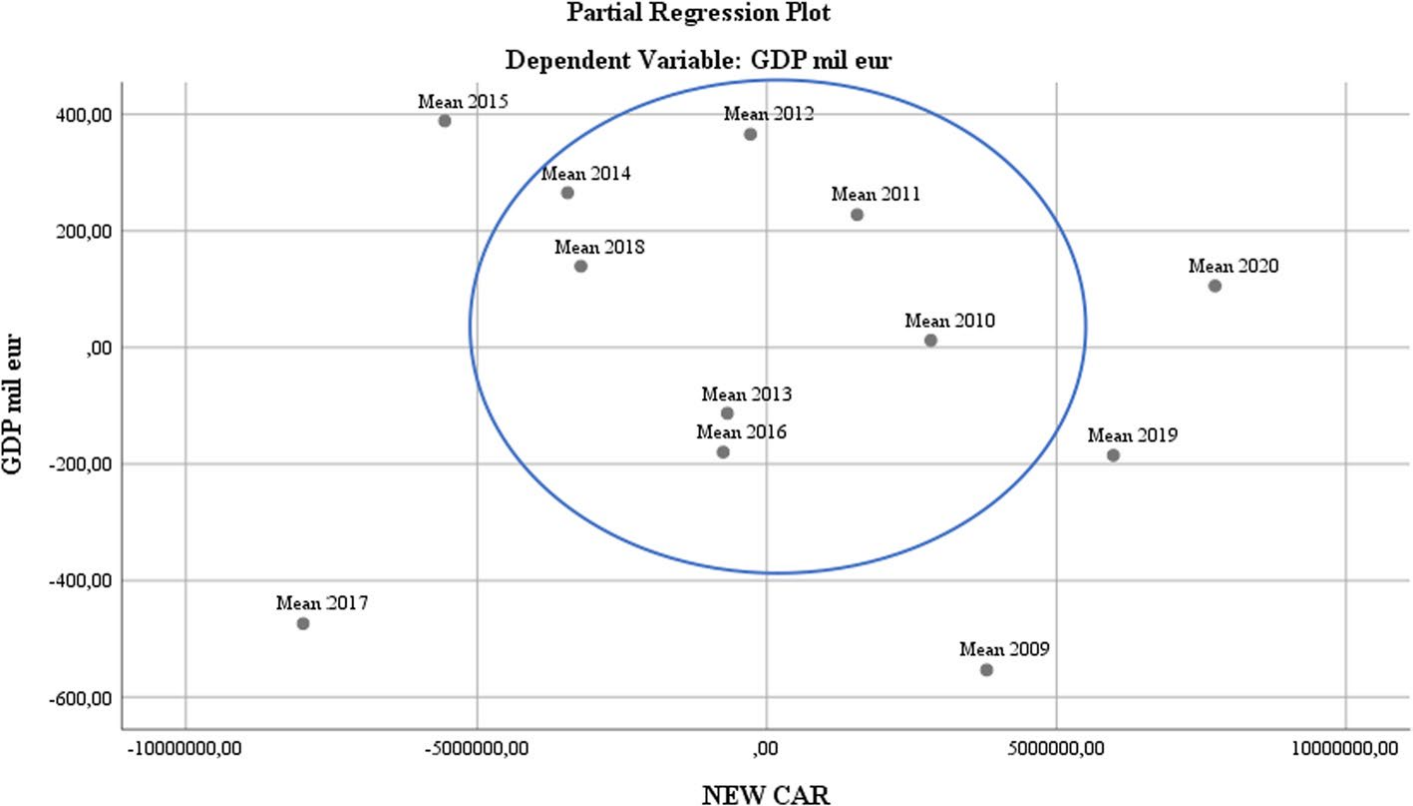
New car regression analysis. *Source*: Author`s contributions

The evolution of the demand for new vehicles at the EU level was consistently high, but the coordinates of the new production of green cars were not felt in 2009. The demand for such vehicles grew exponentially from 2017 to 2019. In 2020, the restrictions imposed by the COVID-19 pandemic changed the structure of transport options to personal ones, increasing the choice of consumers for green vehicles in accordance with the promotion strategy applied by all major car manufacturers (PricewaterhouseCoopers 2020) (see Figs. 9 and 10).

From the presented analysis, the following smart development strategies applicable to cities with the goal of reducing administrative costs and ensuring integrated urban functionality using alternative means generated by the implementation of new technologies and integrated financial flows are as follows (see Table 6):

**Table 6:** Low cost versus expensive costs/solutions

Model indicators	Type	Solutions	Impact	Comments
Local GDP	Low cost implementation	Differentiated charging system; $\mathop {\max }\limits_{ i \to 1} \left( {SMART_{{GDP_{i} }} } \right) \gg GDP_{i} > 0.995$ Progressive system of fines for environmental pollution applied to the local agents; ${ } \cap \left( {{\text{SMART}}_{GDP} ,{\text{NEW}}_{{{\text{CAR}}}} } \right) > 0.652$ Differentiated taxation of the locations which promote RDI. ${ } \cap \left( {{\text{SMART}}_{GDP} ,{\text{RDI}}} \right) > 0.994$	Increasing the collection in the interest of the local community Financing subsequent smart city development projects Development of smart infrastructure projects	Both options are desirable and allow local/regional GDP growth Sustainable development Increasing the quality of life Smart city development
	Expensive solution	Implementation of new technologies in local administration Development of local research for the introduction of new technologies. ${ } \cap \left( {{\text{SMART}}_{GDP} ,{\text{RDI}}} \right) \gg 0.994$ Digitization $\cap \left( {{\text{SMART}}_{GDP} ,{\text{IoT}}} \right) \gg 0.958$	Resource economy Increasing the income of the urban population Increasing the taxpayer satisfaction	
Internet users	Low cost implementation	Supporting the educational policies for access to the new technologies and communications in schools Creating an open society for the implementation of the new technologies $\cap \left( {{\text{IoT}},{\text{RDI}}} \right) > 0.973$	By increasing the number of users on long term Obtaining an educated local population, open to the new	Both options are desirable and allow the sustainability of the process, especially by inserting the new technologies in the school and give the process a high character of sustainable development
	Expensive solution	Creation of urban networking centres Activation of counselling and guidance cells led by specialists/experts Creating IT clusters and developing solutions within them to local problems $\cap \left( {{\text{IoT}},{\text{RDI}}} \right) \gg 0.973$	Immediate accessibility on short and medium term Increasing the degree of adaptation to information technologies for the mature population High possibilities to reduce unemployment and increase the quality of life through information	
RDI	Low cost implementation	Implementation of the 9 stages of technology readiness level in innovation; (Comes et al. 2018) Implementation of the benchmark in innovation ${ } \cap \left( {{\text{RDI}},{\text{IoT}}} \right) > 0.973$	Increasing the applicability of innovation on medium term Connecting theory with practice	Both options are desirable and allow the sustainability of the innovation process and the use of the solutions for the smart city development with an effect in reducing the development costs and immediate operationalization of the measures (also benefiting from the technical support of the specialists who produced the innovation)
	Expensive Solution	Creation of innovation clusters and their operationalization Supporting the RDI process through local administration approaches (with the provision of resources and facilities) ${ } \cap \left( {{\text{RDI}},{\text{Banking}}} \right) \gg 0.998$	Intensifying innovation on medium and short term Creating business opportunities Integration of solutions within the smart city concept	
Internet of Things (IoT)	Low cost implementation	Implementation of IoT at the level of surveillance processes (traffic, waste collection) in the second wave of applications Creating integrated and electronically coordinated flows Ensuring an integrated operational system at the level of local administration for the maintenance of the created flows. ${ } \cap \left( {{\text{IoT}},{\text{NEW}}_{CAR} } \right) > 0.584$	On the medium term, these flows will be operationalized, resulting in a saving of human and material resources at the level of local administration that will exceed the value of the investment during a time period assimilated to a discount rate of 8%	Both variants are desirable; they allow the sustainability of the monitoring process and the implementation of IoT, with effect on the consumption of resources and on the increase of the quality of the local administrative act. (Prince and David 2018)
	Expensive solution	Introduction of teleoperation and telepresence systems with remote object monitoring and control Development of risk sensor detection agent software for IoT’s efficiency and operationalization. ${ } \cap \left( {{\text{IoT}},{\text{Banking}}} \right) \gg 0.975$	On the medium and long term, it will be possible to manage with a very low cost and a maximum limitation of the risks the entire administrative flow at the locality level, which will lead to a maximization of the smart city rate	
Internet banking	Low cost implementation	Multiplication of internet applications for accessing bank accounts and products Stimulating the use of internet banking through various incentives granted to the customers $\cap \left( {{\text{Banking}},{\text{IoT}}} \right) > 0.975$	The increase of internet banking activities on short term The increase of the number of banking internet users on medium and long term Increasing security of the banking operations on short, medium and long term	Both variants are desirable and allow the operational financing of the activities, current economic development, increasing the smart level of local development (smart city)
	Expensive solution	Generalization of the internet banking access through single supervisory mechanism (SSM) and single resolution mechanism (SRM). ${ } \cap \left( {{\text{Banking}},{\text{RDI}}} \right) \gg 0.998$	Increasing the financial security on long term Increasing the speed of accessing funds by shortening the analysis time Supporting the sustainable development	
The overall rate of motorization	Low cost Implementa-tion	Creation of the networks for alternative traffic of cyclists and mopeds Expansion of parks and green spaces. ${ } \cap \left( {{\text{NEW}}_{CAR} ,{\text{SMART}}_{GDP} } \right) > 0.652$	Reducing pollution on short term Traffic reduction Increasing the quality of life	Both variants are desirable, they allow the decrease of pollution, achieving the objectives of sustainable development (environment component) and activating the urban quality indicators related to the smart city
	Expensive solution	Creation of an operational infrastructure and an adequate capacity related to the urban traffic, which should limit the general motorization rate applied in the locality Ensuring optimal power supplies for vehicles $\cap \left( {{\text{NEW}}_{CAR} ,{\text{SMART}}_{GDP} } \right) \gg 0.652$	Reducing pollution on short term Traffic decongestion; Reducing the carbon footprint of the locality; Increasing the quality of life of smart city residents	
Motorized rate of green vehicles	Low cost implementation	Providing power supplies for green vehicles; Application of the differentiated taxation system on the vehicles $\cap \left( {{\text{SMART}}_{GDP} ,{\text{NEW}}_{CAR} } \right) > 0.652$	Increasing the motorization rate of the green vehicles on short term	Both variants are desirable; they allow the achievement of the sustainability objectives on the environment component; Reduction of urban pollution, with long-term effect on increasing the quality of life; Important pillar for smart city development
	Expensive solution	Offering facilities for the construction of services dedicated to green vehicles; Replacement of the local transport fleet with green vehicles. ${ } \cap \left( {{\text{SMART}}_{GDP} ,{\text{NEW}}_{CAR} } \right) \gg 0.652$	Increasing the motorization rate of the green vehicles on long term; Reducing pollution Increasing the quality of life	
Motorization rate of polluting vehicles	Low cost Implementation	Application of the differentiated taxation system on the vehicles; Establishment of areas with access restrictions for polluting vehicles $\cap \left( {{\text{SMART}}_{GDP} ,{\text{NEW}}_{CAR} } \right) > 0.652$	Decrease in the engine rate of polluting vehicles on short term; Reducing pollution; Increasing the quality of life	Both variants are desirable; they allow monitoring the city's fleet of polluting vehicles and limiting it in time Increasing sustainability Increasing the degree of smart city development
	Expensive solution	Establishment of pilot programs to stimulate the replacement of the polluting vehicles (providing facilities) Establishment of a monitoring system of CO_2_ emissions produced by polluting vehicles whose effect should be manifested in strict monitoring of the city's car fleet. $\cap \left( {{\text{SMART}}_{GDP} ,{\text{NEW}}_{CAR} } \right) \gg 0.652$	Decrease in the engine rate of polluting vehicles on medium term**z** Reducing pollution Increasing the quality of life	

According to the two-phase smart implementation Table 6, which covers the proposed objective of piloting a concept implementation tool from accessible to complete, from low cost to expensive solutions, the concept is affordable and implementable not only by cities that know a top economic development. As a result of large urban agglomeration, other cities should carefully monitor the core of indicators whose combined risk affects the quality of life and for which the smart solution is at least a desirable alternative.

By means of the solutions provided in Table 6, we estimate that with their application, there will be an improvement in the Pearson coefficients of the model for the examined cities, and not only the authors estimating the positive effect of the implementation through smart city management, which would become more integrated, easier, and would generate high efficiency and high value-added contribution to the life of the applicant cities’ inhabitants (see objective O4).

The analysis considers that the cheap solutions proposed in the analysis can be implemented in 4–5 years, whereas the expensive solutions require a longer period of time (range: 10–15 years).

Under this research, we achieved the research objectives as follows:

- Identifying an intensification of research in recent years on the smart city concept in the literature, research that highlights the main themes of social, economic, administrative, environmental effects, and urban mobility to improve the quality of life of people living in cities. These concerns are directly linked to the global development objectives promoted through common policies and agendas, which have the ultimate goal of sustainable development and increased population welfare. Therefore, we achieved objective O1 of the research, namely, a critical analysis starting from the current state of scientific knowledge in the field.
- Investigate the dynamics of smart cities–smart community evolution, finding commonalities and major disparities in the development of smart cities. These outputs confirmed that, at the time of the research, there was no unified basis, but only some points of interest on which cities have developed smart approaches, the development being far from complete in the major cities of the world. Accordingly, we have identified several rankings based on the classification of European cities; clearly, the smart city classification is mainly attributed to cities that have a high GDP/capita, that is, they are part of the cluster of large cities in Europe. This approach allows validation of the O2 objective–the development of the smart city/smart community concept– to objectively evaluate the progress of these forms of organization in relation to other classical/traditional forms of town organization.
- Validating our proposed smart model based on the working hypotheses in the Methodology section, which analyzes the effect of adapting smart city/smart community policies at the sample level. This validates objective O3: the transposition of this model by pivoting the hypotheses into a minimal and extended dashboard with pivoting links on each branch of the table facilitates the step-by-step implementation of this form of superior organization of urban populations.
- Identifying two types of solutions (low-cost and expansive) to improve smart urban development applicable to cities whose goal is to reduce administrative costs and ensure integrated urban functionality by using alternative means generated by the implementation of new technologies and integrated financial flows. This allows for the validation of objective O4: the identification of simple solutions, implementable in the short term, up to extensive solutions with high efficiency and high value-added contribution to the lives of the applicant cities’ inhabitants.

## Conclusions

This study addresses maximum interest for the current economy, drawing attention to the need to monitor those indicators of vulnerability that trigger risks in large urban areas and the solution to reduce these risks by developing smart cities.

The four objectives of the research were achieved, with a critical review of the literature (objective O1), including the perspective of smart development models, which helped define the four working hypotheses that were tested through econometric modelling, which was the subject of objectives O2 and O3.

Lastly, based on statistical results that validated the hypotheses after applying one-sided critical probability tests, the authors identified implementable effect solutions for decision-makers and for the promotion of the smart city concept and implementation measures (objective O4). This study presents a novel characteristic, having a high added value owing to the broad component of the research and the design of the econometric models, but also because of the practical solutions provided, which are aimed at helping the smart development of cities.

The outcomes and results of this research consist of defining specific ways of implementing smart development and determining the financial effect of implementing sustainable economic growth on the environment and well-being of citizens. Moreover, the model proposed in this study evaluates the staging of smart city development in relation to the cluster average, highlighting, at the level of the selected indicators, the vulnerabilities that prevent the antipole from aligning with the smart development desideratum.

Our research shows that urban development is directly proportional to R&D efforts and citizens’ ability to use IT infrastructure. The smart equation includes the IoT development and smart governance.

Based on the results, low-cost solutions can be adopted in the short term in terms of local funding levels, increasing IT infrastructure coverage and number of users, RDI development, stimulating interest in IoT, Internet banking, and low-cost solutions for environmental protection, pollution reduction, and the insertion of green technologies in the automotive infrastructure of cities.

These solutions constitute a starting point and a sustainable basis for the implementation of more expensive solutions that would effectively contribute to increasing the intelligence level of cities.

Given the differentiated economic effect, we believe that it is necessary to analyze and implement low-cost solutions at the local government level. These solutions can improve in the short term with immediate effects on the standard of living of the citizens of the cities, but also contribute to the development of the smart city.

The policy implications of this research support a public policy tool for improving the living standards of urban citizens through modern means of upgrading the economic and administrative parameters of urban agglomerations consistent with the new trends promoted through European and global agendas on environmental protection and smart city development.

The main shortcoming of the model is the relatively limited number of indicators; we propose extending the model in a future study, including the implementation of questionnaires at the local administration level of this anti-pole of smart development.

The pathways for further research consist in possibility to extending it to any other large urban agglomeration without translation problems of the model, based on known statistical methods and consolidation principles detailed in this study, and valid demonstrated working hypotheses.

The second post-evaluation stage encompasses identifying possible smart development alternatives for each variable in the monitoring core by building a biphasic picture from accessible to complete, which would give the local administration an effective chance to implement the concept at the locality level.

## Data Availability

Not applicable.

## Abbreviations

CAR: The general motorization rate
COVID-19: Infection with the Coronavirus
DDoS: Distributed denial-of-service
DVR: Digital video recorder
EU: European Union
GDM: Group Decision Making
GDP: Gross Domestic Product
ICT: Information and Communication Technology
IoT: Internet of Things
IT2: Interval type 2
NEWCAR: Motor vehicle rate of green vehicles
OLDCAR: Motorization rate of polluting vehicles
OLS: Least squares method
R&D: Research and development
RDI: Research, Development and Innovation
SEM: Structural equation modelling
SMART: Specific, measurable, attainable, relevant, time-based
SMARTGDP: The smart function of the cities’ economic development based on the transformation of the administrative flows into smart flows
SRM: Single resolution mechanism
SSM: Single supervisory mechanism
TOPSIS: Multiple attribute decision-making problems model
